# The utility of the historical record for assessing the transient climate response to cumulative emissions

**DOI:** 10.1098/rsta.2016.0449

**Published:** 2018-04-02

**Authors:** Richard J. Millar, Pierre Friedlingstein

**Affiliations:** 1 College of Engineering, Mathematical and Physical Sciences, University of Exeter, Exeter, UK; 2 Environmental Change Institute, University of Oxford, Oxford, UK

**Keywords:** climate change, carbon cycle, carbon budgets, Paris Agreement

## Abstract

The historical observational record offers a way to constrain the relationship between cumulative carbon dioxide emissions and global mean warming. We use a standard detection and attribution technique, along with observational uncertainties to estimate the all-forcing or ‘effective’ transient climate response to cumulative emissions (TCRE) from the observational record. Accounting for observational uncertainty and uncertainty in historical non-CO_2_ radiative forcing gives a best-estimate from the historical record of 1.84°C/TtC (1.43–2.37°C/TtC 5–95% uncertainty) for the effective TCRE and 1.31°C/TtC (0.88–2.60°C/TtC 5–95% uncertainty) for the CO_2_-only TCRE. While the best-estimate TCRE lies in the lower half of the IPCC likely range, the high upper bound is associated with the not-ruled-out possibility of a strongly negative aerosol forcing. Earth System Models have a higher effective TCRE range when compared like-for-like with the observations over the historical period, associated in part with a slight underestimate of diagnosed cumulative emissions relative to the observational best-estimate, a larger ensemble mean-simulated CO_2_-induced warming, and rapid post-2000 non-CO_2_ warming in some ensemble members.

This article is part of the theme issue ‘The Paris Agreement: understanding the physical and social challenges for a warming world of 1.5°C above pre-industrial levels'.

## 1. Introduction

The use of Earth System Models (ESMs), full complexity climate models which explicitly simulate important biogeochemical cycles (such as carbon) in the climate system, have enabled an improved understanding of the link between emissions of climatically important gases and realized climate change [1,2]. As atmospheric carbon dioxide (CO_2_) concentration anomalies have long decay timescales [3], CO_2_-induced warming is essentially permanent on human-relevant timescales [4,5] without the deployment of CO_2_ removal technologies. Understanding possible future changes in the coupled climate–carbon cycle are therefore of critical importance to making long-term projections of climate change over centuries to millennia [6–8].

ESM simulations have helped quantify the gradient of the approximately linear and scenario-independent relationship between cumulative emissions of CO_2_ and resultant global mean warming [9–12] through coordinated experiments run by a set of ESMs from around the world [2]. Using ESM-based estimates along with observational constraints, the 5th Assessment Report (AR5) of the Intergovernmental Panel on Climate Change (IPCC) assessed the *transient climate response to cumulative emissions* (TCRE—the global mean warming following a 1 TtC (1000 GtC) injection of CO_2_ into the atmosphere) to be *likely* (greater than 66% probability) between 0.8 and 2.5°C [13], similar to the 0.8–2.4°C ESM-based TCRE range reported in [2].

The value of the TCRE has direct relevance to contemporary international climate policy as the approximate linearity between warming and cumulative emissions leads to an all-time ‘budget’ of allowable cumulative CO_2_ emissions compatible with any given warming threshold, including the 1.5°C and 2°C thresholds of the Paris Agreement long-term temperature goal [14,15]. While the timing of greenhouse gas (GHG) emission reductions is important for economically efficient implementations of climate policy, particularly at national or regional levels, the cumulative CO_2_ budget gives an unavoidable physical constraint on the total global CO_2_ emissions over all time. Although partitioning the global carbon budget into national shares is complex and value laden [16–18], carbon budget framings may play an important role in setting national-level policies to meet the long-term temperature goal of the Paris Agreement.

The large uncertainty in estimates of TCRE propagates into substantial uncertainty in the allowable carbon budget for a given temperature threshold. For instance, varying TCRE from 0.8 to 2.5°C would change the all-time CO_2_-only cumulative carbon budget for a 2°C threshold (which would only correspond to an absolute warming of 2°C from preindustrial under the assumption that net non-CO_2_ warming is negligible at time of peak warming) by 1700 GtC (ranging from 800 to 2500 GtC), corresponding to over 150 years of present-day global CO_2_ emissions.

Given the large uncertainties in TCRE arising from ESM simulations, this paper investigates to what extent useful information can be derived from the historical record to further inform estimates of the TCRE, and hence constrain estimates of remaining carbon budgets. We build on the observational estimates made in both [9] and [2] to provide an update on observationally constrained TCRE, incorporating the most recent observations of both warming and emissions. The observational and model data used are discussed in §2. In §3a, we use a detection and attribution method that does not directly rely on estimates of forcing or response from complex climate models to estimate the all-forcing or ‘effective’ TCRE (the global mean warming from all-forcing agents following 1 TtC cumulative CO_2_ emissions) [19] from observations and up-to-date assessments of radiative forcing uncertainty. We then compare these observational effective TCRE estimates with those simulated over the historical period in ESMs. In §3b, we discuss inferences about uncertainty in the CO_2_-only TCRE over the historical period and compare with estimates of ESM-simulated historical CO_2_-induced and non-CO_2_-induced. Section 4 contains a concluding discussion.

## 2. Data and methods

### (a) Observational data

Observational temperature time-series are taken from a spatially in-filled version of the Met Office Hadley Centre HadCRUT4 dataset [20] (which we refer to as HadCRUT4-CW). This 100 member ensemble of hybrid air/sea temperatures uses a statistical technique to in-fill unobserved regions in the original HadCRUT4 product [21] so as to achieve complete global coverage and enable a like-for-like comparison with global ESM output [22]. Observational data are used at annual time resolution up until the end of 2016. The HadCRUT4-CW ensemble allows for a systematic sampling of observational uncertainty; however, methodological choices between different observational product represent another source of uncertainty. As a sensitivity test, we also conduct analysis using the best-estimate warming from the Berkeley Earth dataset [23], which shows higher warming over the historical period (see the electronic supplementary material).

Observed cumulative emission estimates are taken from the 2016 Global Carbon Project (GCP) dataset [24]. Observational uncertainties are accounted for using an ensemble created by independent sampling of uncertainties in fossil fuel and industry emissions, and land-use emissions as described in [24]. We also conduct sensitivity analysis using the 2017 GCP dataset [25], which indicates substantially higher historical land-use change emissions (see the electronic supplementary material).

A history of effective radiative forcing (ERF), and its uncertainty, is required to estimate the fraction of observed warming associated with both human activities and that associated specifically with CO_2_ emissions only. Best-estimate time-series of historical ERF for the individual components of anthropogenic and natural radiative forcing (e.g. CO_2_, other well-mixed GHGs, aerosols and volcanic) are taken from Myhre *et al.* [26] and have been extended using observational data up to the end of 2016 as in [27]. The central estimate of ERF from non-CO_2_ GHGs has been updated to incorporate a recent upward revision to the methane radiative forcing proposed in [28]. Volcanic forcing for the recent period is taken from Andersson *et al.* [29], and solar forcing using data from Kopp [30].

Historical ERF uncertainty is sampled by multiplying the best-estimate forcing time-series for a given component of ERF by draws from a distribution of the fractional uncertainty in that component's 2011 forcing, as assessed in IPCC-AR5 [26]. Individual forcing components are sampled independently and then combined to create a distribution of uncertainty in total historical ERF on the climate system (see electronic supplementary material, figure S1).

### (b) ESM simulations

The ESM data used in this paper are taken from experiments conducted under the 5th Coupled Model Intercomparison Project—CMIP5 [31]. We use results from the historical simulation as well as from the Representative Concentration Pathway (RCP) scenarios [32,33] and the idealized 1%/yr atmospheric CO_2_ concentration increase experiment [34]. The list of ESMs used in this analysis is as follows: CESM1-BGC, CanESM2, GFDL-ESM2G, GFDL-ESM2M, HadGEM2-CC, HadGEM2-ES, IPSL-CM5A-LR, IPSL-CM5A-MR, IPSL-CM5B-LR, MIROC-ESM-CHEM, MIROC-EMS, MPI-ESM-LR, NorESM1-ME, bcc-csm1–1 and inmcm4. Models for which data are not available for the 1%/yr experiment are omitted from analysis that involves the use of those experiments (§3b(i)). In comparing the ESMs against the observational record, we consider the effect of observational processing on the modelled temperature anomalies to allow a like-for-like comparison with observational products. ‘Blended’ time-series of globally complete simulated warming are taken from [22], which processed model output using a blend of surface air-temperatures (over land) and sea-surface temperatures (over ocean), similar to as in observational products. This enables a direct ‘like-for-like’ comparison between globally complete model output and observational products that are statistically in-filled to address observational coverage gaps in order to estimate a global mean value. We use the RCP8.5 scenario [35] when comparing ESMs to observations over the post-2005 period.

Diagnosed ESM CO_2_ emissions from Jones *et al.* [33] (historical and RCP experiments) and Gillett *et al.* [2] (1%/yr experiment) are used throughout. Land-use emissions have been added, where required, identically to as in [36]. Time-series of both warming and cumulative emissions for all RCP ESM simulations are smoothed with a 10 year centred moving average to remove modes of unforced natural variability and help isolate the externally forced signal (1%/yr ESM simulations are smoothed with a 15 year running mean).

## 3. Results

### (a) Estimating effective TCRE from the observational record and ESMs

Observed warming is driven by multiple anthropogenic forcing agents; however, the concept of TCRE was derived from model-based results that focused primarily on CO_2_-only forced simulations [2,9]. The physical mechanisms that give rise to the approximate linearity between CO_2_-induced warming and cumulative emissions, namely the compensation between logarithmic CO_2_ radiative forcing and increasing airborne fraction [9,37], apply to CO_2_-induced warming only. As such, a time-independent effective (all-forcing) TCRE would only be expected to the extent that CO_2_-induced warming dominates the forcing mix, or if the relative contribution of non-CO_2_ forcing to warming remains approximately constant over time.

**Figure 1.**
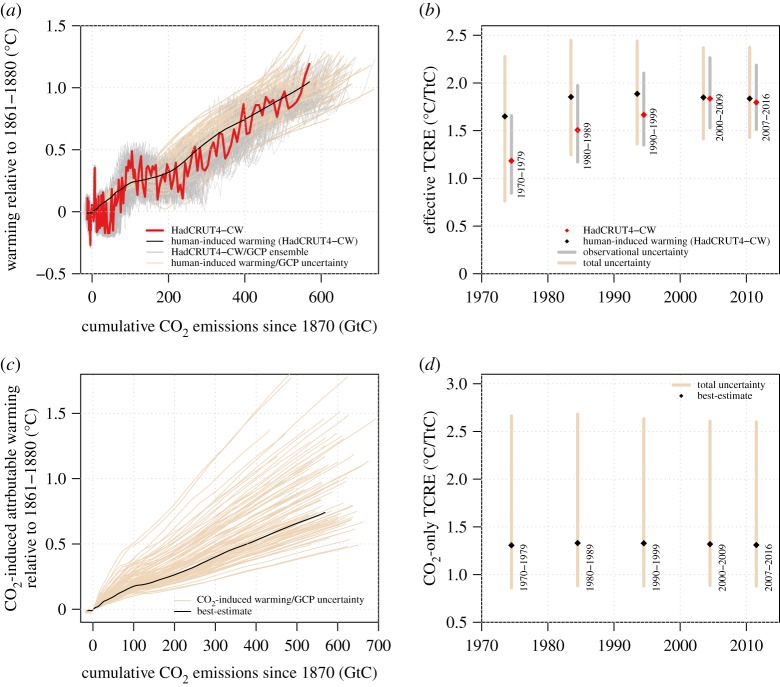
Implications of the historical record for constraining TCRE. (*a*) Time-series of warming as a function of cumulative emissions for members of the observational HadCRUT4/GCP ensemble (grey), the best-estimate HadCRUT4-CW/GCP time-series (red) and best-estimate human-induced warming for HadCRUT4-CW (black) with beige lines showing a random sample from the joint human-induced warming/emission uncertainty. Estimates for the effective TCRE over a range of periods are shown in (*b*), derived from the same data as in (*a*). Grey bars show 5–95% ranges for observational uncertainty from the HadCRUT4/GCP ensemble. Beige bars show 5–95% ranges for total uncertainty from the human-induced warming calculation and emission observations. (*c*) The best-estimate (black line) and a random sample from the joint evolution of CO_2_-induced warming and cumulative emissions over the historical period (beige lines). Panel (*d*) as for panel (*b*) but using the data in panel (*c*). Black dots mark the best-estimate CO_2_-only TCRE estimate for each period. (Online version in colour.)

Observational time-series (together with their uncertainties, figure 1*a*) can be used to assess the extent that the effective TCRE varies over time in the observational record. Grey bars in figure 1*b* show the 5–95% of historical effective TCRE associated purely with observational uncertainty. These are estimated using independent pairings of the HadCRUT4-CW and historical cumulative emission ensembles averaged over the periods shown to the right of the bars. They show a non-constant effective TCRE increasing from the early 1970s up until the 2010s. For the most recent decade (2007–2016), purely observational uncertainty in effective TCRE spans a 1.51–2.19°C/TtC 5–95% range, dominated by uncertainty in the historical cumulative emissions which vary by ±17% (5–95% uncertainty) compared with ±8% for the HadCRUT4-CW ensemble.

Observed warming is determined by a combination of an externally forced warming (associated with human and natural forcing on the climate system) and natural climate variability. Unlike contributions from natural variability, warming driven by human activity is expected to evolve relatively smoothly in time. To estimate human-induced warming, we use an attribution analysis. This analysis is based on the optimal fingerprinting method of detection and attribution of climate change [38], which uses a multiple linear regression to decompose observed warming, Δ*T*_obs_, into a forced component associated with anthropogenic drivers, Δ*T*_anthro_—the human-induced warming, a forced component associated with natural drivers, Δ*T*_nat_, and an unforced residual natural variability (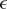), with *δ* acting as a regression constant,
3.1
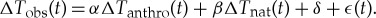

**Figure 2.**
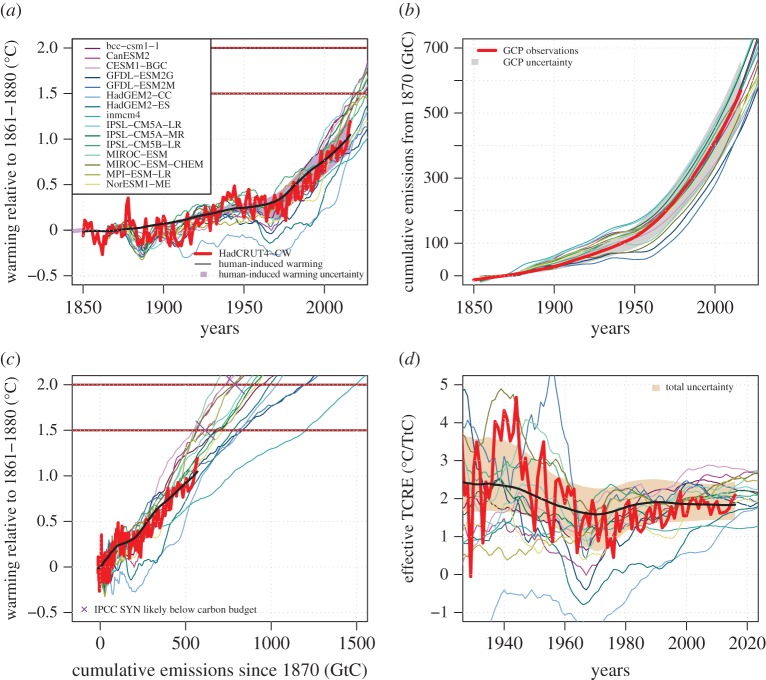
Comparison of ESM-simulated warming (blended) and diagnosed cumulative CO_2_ emissions (thin pastel coloured lines) with unsmoothed in-filled observations (HadCRUT4-CW/GCP—thick red) and its attributed human-induced component (black—with 5–95% shading shown in purple in (*a*)). (*a*) Warming relative to the average of 1861–1880, (*b*) diagnosed cumulative CO_2_ emissions since 1870 (GCP observations are shown in red with grey shading 5–95%), (*c*) the joint evolution of warming and cumulative CO_2_ emissions, and in (*d*) the all-forcing effective TCRE calculated with a moving average. The black line in (*c*) and (*d*) uses the attributed human-induced warming time-series from (*a*). ESM temperature time-series are ‘blended’ air/sea warming smoothed using a 10 year centred running mean in all cases. Beige shading in (*d*) indicates the total uncertainty from the calculation of human-induced warming and historical emissions. The purple crosses in (*c*) mark the all-time likely below carbon budget from the IPCC-AR5 Synthesis Report (IPCC SYN). (Online version in colour.)

We use a two-time constant impulse response model to simulate the forced warming regressor, Δ*T_n_*, for a particular component, *F_n_*(*t*), of the total ERF acting on the climate system [39,40],
3.2
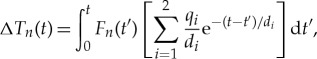

where impulse response model parameters, *q_i_* and *d_i_*, represent parameters of the climate response to radiative forcing. Using an impulse response model to estimate Δ*T*_anthro_(*t*) and Δ*T*_nat_(*t*), as opposed to using simulations from the CMIP5 archive, allows the latest revisions in estimates of historical ERF components, and a broader range of possible fractional contributions of CO_2_ to net forcing than simulated in the CMIP5 ensemble, to be used when making inferences about TCRE over the historical period. We account for uncertainty in the historical ERF using a distribution of possible ERF histories (see §2a) and parametric uncertainty using a sampling of *q_i_* and *d_i_* consistent with distributions for the realized warming fraction (the ratio of transient climate response—TCR—to equilibrium climate sensitivity [40]) and the short thermal response timescale of the climate system from Millar *et al.* [41], approximating the distributions of these variables across CMIP5 general circulation models. Observational uncertainty is sampled using the HadCRUT4-CW ensemble and uncertainty associated with natural climate variability is sampled by adding realizations of control climate variability from CMIP5 to the observed time-series. Total uncertainty (5–95%) in attributed human-induced warming is shown by the purple shading in figure 2*a*. Haustein *et al.* [27] showed that the dominant sources of uncertainty in this calculation are ERF and internal variability uncertainties. As control variability is added using global surface air-temperature fields, this may somewhat over-estimate the contribution of internal variability uncertainty in the blended air-temperature/sea-surface temperature product.

The effective TCRE calculated using human-induced warming (black in figure 1) displays substantially less variation over time than estimates from pure observed warming (grey bars) as the changing contributions of both temporary natural forcing (such as volcanic eruptions) and natural climate variability have been regressed out. This indicates that natural climate variability typically has a larger impact on estimates of effective TCRE from observations than changing fractional contributions to forced warming associated with evolving historical non-CO_2_ forcing. Total uncertainty in the attributed effective TCRE is larger (beige bars in figure 1*b*) than that associated with purely observational uncertainty, as decadal mean uncertainty is much larger in human-attributable warming than observed warming. Over the 2007–2016 period, the total uncertainty on the attributed effective TCRE displays a 5–95% range of 1.43–2.37°C/TtC and a best-estimate of 1.84°C/TtC.

#### (i) Effective TCRE in ESMs

As CMIP5 ESM simulations were driven by observed atmospheric CO_2_ concentration pathways, compatible anthropogenic emissions can be diagnosed from the simulated land and ocean carbon sinks compatible with the prescribed increase in atmospheric CO_2_ [33]. Therefore, CMIP5 historical simulations can be explicitly validated against the observational record in terms of their joint simulation of warming and anthropogenic cumulative CO_2_ emissions, as both of these variables are outputs of the ESM simulations.

Comparisons between the observed record and ESMs for temperature and diagnosed cumulative CO_2_ emissions are shown in figure 2*a*,*b*. In 2016, the ensemble mean of the smoothed global mean blended air/sea warming for the ESMs (solid thin coloured lines) was 0.10°C (0.41 to −0.27°C ensemble range) warmer than the estimated human-induced warming from global (HadCRUT4-CW) observations (solid black line approximately 1°C in 2016). Historical cumulative CO_2_ emissions (to the end of 2016) are typically underdiagnosed relative to the GCP observations in many of the ESMs (by 27 GtC for the multi-model mean, 121 to −65.7 GtC ensemble range), with nine out of 15 ESMs diagnosing historical cumulative emissions to be less than best-estimate observed emissions between 1870 and 2016. As many ESMs typically underestimate land or ocean carbon sinks, and hence compatible annual anthropogenic CO_2_ emissions needed to close the carbon budget [6,33,42,43], such underestimates in historical cumulative emissions are expected. This underestimate of cumulative emissions in ESMs relative to the best-estimate observed value is persistent over the last half century and as such cannot be attributed to an artefact of the so-called ‘hiatus’ in warming which could have acted to temporarily reduce the observed annual airborne fraction, therefore allowing models to meet a similar concentration evolution with lower diagnosed emissions.

Combining the ESM simulations of warming and diagnosed cumulative emissions typically produces estimates of the effective TCRE greater than the best-estimate from the historical period (figure 2*c*,*d*). Subsequent to substantial variability in the effective TCRE prior to 1980, in both ESMs and observations the effective TCRE has been approximately constant since 2000. ESMs show a higher value than estimated from the observational best-estimate in 12 out of 15 ESMs by 2016 (figure 1*d*). Despite the substantial variation between different observationally based estimates of the effective TCRE, and substantial uncertainty in human-induced warming, the mean ESM effective TCRE (2016) is 0.27°C/TtC higher than the estimate based on attributable human-induced warming, and four out of 15 ESMs have effective TCREs greater than the observational 95th percentile for 2016 (approx. 2.45°C/TtC; figure 2*d*).

**Figure 3.**
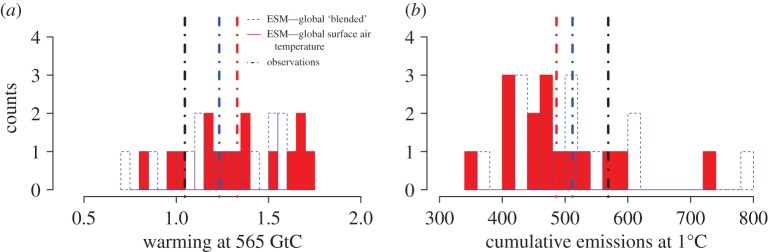
ESM estimates of warming after 565 GtC of diagnosed cumulative CO_2_ emissions (*a*) and diagnosed cumulative CO_2_ emissions after 1.0°C of simulated warming (*b*). Filled red bars show results calculated with full global coverage surface air-temperature warming and dashed blue outlines show blended ESM output. Dotted-dashed red and blue vertical lines indicate ESM ensemble means (for global and global blended outputs) and dotted-dashed black vertical lines indicate the observations, the human-attributable component of observed warming from HadCRUT4-CW in (*a*) and the GCP cumulative emissions in (*b*). (Online version in colour.)

Remaining carbon budgets estimates from ESMs are determined by the coevolution of simulated warming and diagnosed CO_2_ emissions and not by either in isolation. All but three of the 15 ESMs considered here have a greater warming than observational best-estimate human-induced warming when diagnosed cumulative emissions reach 565 GtC in both the blended and global surface air-temperature ESM time-series, with some models displaying warming already in excess of 1.5°C for 565 GtC emissions (figure 3*a*) for both types of model output. Similarly, both blended and global surface air-temperature ESM time-series can have substantially lower diagnosed cumulative emissions than the observed best-estimate when warming reaches the 2016 best-estimate human-induced value of approximately 1.0°C (figure 3*b*).

### (b) Estimating CO_2_-only TCRE

Estimating the CO_2_-only TCRE from observations requires an estimate of the fraction of human-induced warming that is attributed to CO_2_. While CO_2_ radiative forcing is relatively well constrained, total anthropogenic forcing remains uncertain largely due the range of possible aerosol ERF once interactions with clouds are accounted for [26,44,45]. For a given member of the ERF uncertainty sampling (subscript *i*), linear regression (equation (3.1)) can be used to decompose attributed human-induced warming, 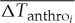 (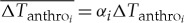), into the forced warming associated with each component of the anthropogenic radiative forcing,
3.3
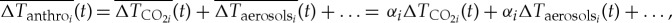

where responses to the sampled individual components of the forcing (e.g. 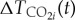) are computed using equation (3.2). Convolving independent sampling of ERF uncertainty and other uncertainties, as described in §3a, provides a distribution for 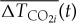 that can be combined with uncertainty in historical cumulative CO_2_ emissions to make inferences about the CO_2_-only TCRE from observations.

The large uncertainty in aerosol ERF propagates into a wide range of possible contributions of CO_2_ to observed warming today. Figure 1*c* shows 300 members of the joint distribution of CO_2_-induced warming and cumulative emissions (beige lines) with the best-estimate marked with the black line. The estimated CO_2_-induced warming to-date (2016) ranges from as little as 0.51°C (5th percentile) to as much as 1.43°C (95th percentile) respectively, representing extremal cases in which non-CO_2_ greenhouse gas forcing strongly dominates a weakly negative aerosol forcing (5th percentile) and when net non-CO_2_ forcing is itself negative due to a strong negative aerosol forcing, partly masking the CO_2_-induced warming in the present-day climate (95th percentile). As long-lived forcing on the climate system is largely associated with elevated CO_2_ and N_2_O concentrations (N_2_O currently contributes only approx. 9% of the forcing associated with CO_2_ in the best-estimate case), our results indicate that there is a low, but non-zero, chance that current warming from long-lived GHGs already exceeds 1.5°C based on current-forcing uncertainties. This indicates that it still remains relatively unlikely that we are yet geo-physically locked-in to a long-lived component of warming in excess of the high ambition goal of the Paris Agreement and therefore a need to use carbon removal or solar geoengineering techniques to limit long-term warming beneath this threshold.

The best-estimate of CO_2_-induced warming in 2016 is 0.74°C, corresponding to the best-estimate radiative forcing components, in which CO_2_ forcing represents 74% of the net forcing in 2016. Approximately linear relationships between cumulative emissions and attributed CO_2_-induced warming are seen across the uncertainty range from the attribution analysis (figure 1*c*), leading to approximately period-invariant best-estimates and uncertainty intervals on the implied CO_2_-only TCRE estimates (figure 1*d*). The best-estimate CO_2_-only TCRE estimate over the most recent decade (2007–2016) is 1.31°C/TtC and is approximately invariant over the four previous decades considered here. The 5–95% range of the TCRE over the most recent decade is 0.88–2.60°C/TtC, extending beyond the upper end of the 0.8–2.5°C/TtC *likely* range of TCRE assessed by AR5. The overall similarity between the two ranges might be expected due to the inclusion of observational constraints in the expert judgement underlying the AR5 assessment. While observational attribution supports a best-estimate CO_2_-only TCRE in the bottom half of the AR5 likely range, the uncertainties associated with historical forcing mean that uncertainties in the observationally estimated CO_2_-only TCRE remain large, and a high TCRE cannot be conclusively ruled out by current observations.

#### (i) ESM-simulated historical CO_2_-induced and non-CO_2_-induced warming

Using a combination of historical simulations and idealized 1%/yr CO_2_ concentration increase experiments, it is possible to partition simulated historical warming in ESMs into CO_2_-induced and non-CO_2_-induced components. From a time-series of simulated warming under the 1%/yr experiments for a given ESM, it is possible to estimate the CO_2_-induced warming for a given level of cumulative CO_2_ emissions. Comparisons with the estimate of the simulated warming in the historical simulations for the same level of cumulative emissions allows a quantification of the warming induced by CO_2_ alone versus the warming from non-CO_2_ anthropogenic and natural forcing components.

This method assumes a linear separability between CO_2_ and non-CO_2_ warming, and a perfect scenario independence of the relationship between cumulative CO_2_ emissions and CO_2_-induced warming. While evidence exists for dependence of carbon-cycle feedbacks on the total warming within the climate system [46,47], as well as some scenario dependence of the response to cumulative emissions [48], to a first order these remain good assumptions to partition ESM-simulated historical warming into CO_2_ and non-CO_2_ contributions. An additional implicit assumption is that carbon-cycle feedbacks (which give rise to the relationship between warming and cumulative emissions in these experiments) estimated from the 1%/yr simulation are a good proxy for the carbon-cycle feedbacks over the historical period. For example, as the 1%/yr simulations have fixed preindustrial land-use forcing, unlike the historical simulation, some differences might be expected in the land carbon feedbacks between the two experiments for a single climate model. We provide a proxy test of the similarity between ESM carbon-cycle feedbacks in the two experiments using a simple climate carbon-cycle model in the electronic supplementary material. Based on this technique, we conclude that the difference in carbon-cycle feedbacks between the two experiments is unlikely to have a large effect on the diagnosed cumulative emissions over the historical integration, but could be expected to have a substantial effect under high future emission scenarios such as RCP8.5.

Diagnosed cumulative emissions for a given atmospheric concentration evolution will also somewhat depend on the profile of other forcing agents, due to both non-CO_2_ contributions to warming affecting natural carbon sinks and (in the case of aerosols) changes to the biogeochemical carbon-cycle feedback by direct impact on diffuse surface radiation. MacDougall *et al.* [49] showed that in the UVic intermediate complexity model, the inclusion of non-CO_2_ radiative forcing only caused a small reduction in diagnosed cumulative CO_2_ emissions over the historical period. Similarly, the study of MacDougall & Knutti [50] indicates only a fractionally small increase in long-lived warming associated with the effect of present-day methane emission rates on the efficacy of carbon sinks. Therefore, while the effects of both non-CO_2_ forcing on carbon sinks, and possible difference between carbon-cycle feedbacks in the 1%/yr and historical integrations, add uncertainty to estimates of ESM CO_2_-induced warming, the method used here can serve as a useful approximation over the historical period.

**Figure 4.**
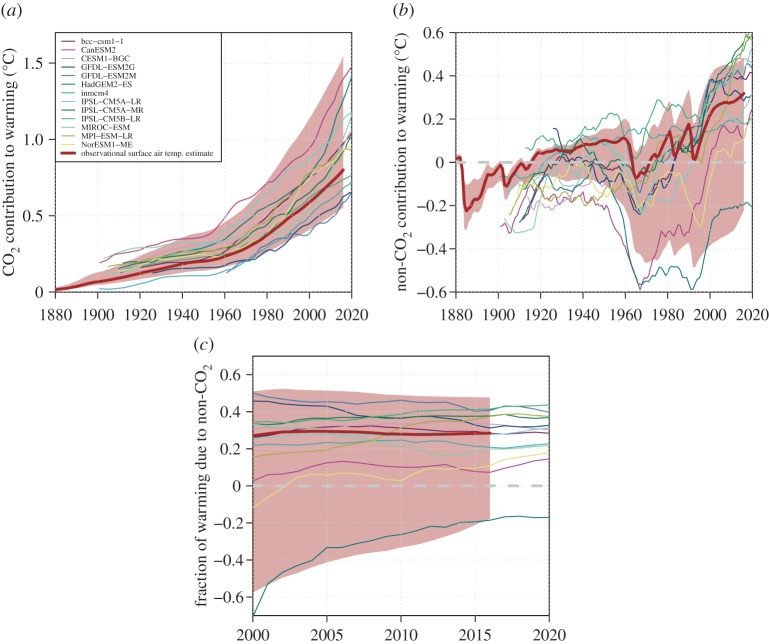
Historical CO_2_-induced and non-CO_2_-induced global surface air-temperature warming. ESMs (global mean air-temperature warming) are shown with thin coloured lines and estimated values from the observational attribution with a thick brown line (best-estimate—brown shading indicates 5–95% uncertainty). The observational estimate has been ‘de-blended’ (to estimate observed global mean surface air-temperature warming compatible with the blended air/ocean temperature observational series) to allow a like-for-like comparison with global mean air-temperature warming in the 1%/yr simulation from the ESMs. (*a*) Estimated CO_2_-induced warming and (*b*) estimated non-CO_2_ anthropogenic and naturally forced warming. (*c*) The fraction of total warming due to non-CO_2_ drivers for the post-2000 period. (Online version in colour.)

ESM-derived CO_2_-induced air-temperature warming evolves steadily over time (figure 4*a*) and reaches a mean value of 0.93°C in 2016 (0.59–1.40°C 5–95% range), 0.13°C higher than the best-estimate observed surface air-temperature warming (which we calculate by ‘de-blending’ the observational product using the CMIP5 mean ratio between global air-temperature and blended air/ocean warming from Richardson *et al.* [51]). The ESM simulations span a range of CO_2_-induced warming consistent with uncertainties in the estimated attributable CO_2_-induced warming from observations. This similarity would be expected due to a similar range of TCRE uncertainty in attributed CO_2_ warming and the ESM 1%/yr experiments.

Non-CO_2_-simulated warming in the ESMs (figure 4*b*) has an ensemble mean value approximately equal to the observationally based best-estimate, while three out of 11 ESMs for which CO_2_ and non-CO_2_ contributions to warming are calculable have non-CO_2_ warming greater than the 95th percentile of the attribution-estimated contribution in 2016, with some ESMs displaying a sharp increase in non-CO_2_ warming around 2005 (the start of the RCP scenarios). Non-CO_2_-induced warming as a fraction of total warming has remained relatively stable in both ESMs and as estimated from observations since 2000 (figure 4*c*), with a recent uptick observed in the observational best-estimate largely associated with an increase in methane radiative forcing over the last decade.

## 4. Discussion and conclusion

A recent study [52] used a scenario-based approach to propose a larger remaining carbon budget for limiting warming to below 1.5°C than the IPCC-AR5 assessment [15]. This was based, in part, on applying ranges of ESM-based uncertainty in the warming response to cumulative CO_2_ emissions to deduce remaining budgets for further warming above the present-day climate state, but did not try to further constrain the TCRE or effective TCRE from the ESM range. Here, we have analysed the implications of the observational record of warming and cumulative CO_2_ emissions to update previous observational constraints on the TCRE. While uncertainties associated with natural variability and poorly constrained ERF of non-CO_2_ climate forcers are generally large, we find a best-estimate TCRE from an attribution analysis of the historical record of 1.31 K/TtC, in the lower half of the IPCC-AR5 likely range, with uncertainties approximately spanning the 0.8–2.5 K/TtC AR5 TCRE range (0.88–2.60°C/TtC 5–95% range). We find a higher upper uncertainty bound on TCRE than found using observational constraints in [2], possibly associated with the different methodologies for accounting for uncertainty in the fractional contribution of CO_2_-induced warming to total forced warming. Differences over the historical period between ESMs and observations seem to arise in part from a higher mean CO_2_-induced warming in the ESM ensemble, rapid non-CO_2_ warming since 2000 in some ESMs, and from lower than observed diagnosed cumulative CO_2_ emissions for many members of the ESM ensemble.

Based on our best-estimate of the historical effective TCRE (1.84°C/TtC), assuming a constant effective TCRE into the future would be compatible with a best-estimate 520 GtC remaining budget (approx. 47 years of current emissions—all budgets given to nearest 5 GtC) for 2°C and 250 GtC (approx. 23 years of current emissions) for 1.5°C. Using global air-temperature warming from ESM simulations only, IPCC-AR5 assessed that limiting warming below 2°C relative to preindustrial, with a probability greater than 66%, requires cumulative carbon emissions (since preindustrial) to remain below 790 GtC (top purple cross in figure 1*c*). AR5 Synthesis Report [15] provided a 615 GtC all-time budget for remaining below 1.5°C in 66% of CMIP5 RCP8.5 simulations (assuming a constant effective TCRE compatible with the 2°C budget would imply a 1.5°C all-time budget of 590 GtC). Subtracting the latest estimates of historical emissions, updated to 2016 inclusive (565 GtC, [24]), the remaining cumulative carbon emissions consistent with the 1.5°C and 2°C target could be inferred as 50 and 225 GtC, respectively, equivalent to approximately 5 and 20 years of present-day emissions. As the ESM distribution lies to the top end or even outside of the uncertainty distribution for the historical effective TCRE, estimates of the carbon budget to keep warming likely below 1.5°C based on the upper end of the ESM effective TCRE distribution may be overly restrictive.

The observational uncertainties that we consider here do not include the effect of methodological choices for both historical warming and cumulative emissions, which can be large. As a sensitivity exercise we use the Berkeley Earth temperature dataset [23] (which displays a larger warming from preindustrial than HadCRUT4-CW), and the recently released GCP 2017 dataset [25], which includes a substantial increase in the best-estimate of historical land-use change CO_2_ emissions. Using the Berkeley Earth temperature dataset (electronic supplementary material, figure S5) would increase the estimated effective TCRE best-estimate to 2.05°C/TtC (1.46°C/TtC for the CO_2_-only TCRE), while using the 2017 GCP dataset (electronic supplementary material, figure S6) would reduce the effective TCRE best-estimate to 1.72°C/TtC (1.23°C/TtC for CO_2_-only TCRE), with widened uncertainty bounds associated with greater uncertainty over historical land-use change emissions.

There are several reasons to be cautious about extrapolating carbon budgets from effective TCRE estimates derived from historical data alone. Inferences from the historical period may not be particularly useful for understanding climate changes under very high future emission scenarios. Climate feedbacks may evolve substantially over time [53], leading to increases in important climate response parameters such as the transient climate response beyond the first doubling of CO_2_ [54]. However, for very ambitious mitigation scenarios, changes in the transient climate response and the effect of additional carbon-cycle feedbacks on remaining budgets would be expected to be much smaller than seen under high future emission scenarios. Therefore, if risks of ‘tipping-point’ Earth system feedbacks below 2°C are small, the historical record could indeed offer relevant information about the TCRE that is useful for assessments of pathways to meet ambitious mitigation goals. However, the risk of currently unpredicted and unmodelled Earth system feedbacks still remains possible and acts as a caveat on all inferences about remaining carbon budgets from the historical period. The probability of additional Earth system feedback, and its impact on remaining carbon budgets, should be a topic for further scientific investigation.

An additional important caveat regarding the use of the historical record to constrain remaining carbon budgets is the extent to which the future relationships between CO_2_-induced warming, non-CO_2_-induced warming and natural variability are likely to change. As emissions of CO_2_ and other climatically important gases are co-emitted in many cases, it remains unclear to the extent that CO_2_ and non-CO_2_ mitigation can be considered separable at a global scale, given uncertainties in future technological development and economic demand [55]. Rogelj *et al.* [56] indicates that future emission scenarios span a wide range of best-estimate contributions from non-CO_2_ warming at the time of crossing temperature thresholds, which, when convolved with the physical uncertainties for a specific emission scenario (see electronic supplementary material, figure S4), leads to substantial uncertainty regarding the relative changes in the fractional contribution of non-CO_2_ warming to temperature thresholds.

If future non-CO_2_ mitigation could be implemented to reduce the fraction of non-CO_2_ warming below today's levels by the time of net-zero CO_2_ emissions, then remaining budgets would be further extended. For example, if this fraction could be reduced to approximately 15% at peak warming, our best-estimate remaining budgets for 1.5°C and 2°C may be extended to approximately 730 and 410 GtC, respectively (based on the best-estimate CO_2_-only TCRE), indicating the potential importance of this uncertainty for ambitious mitigation policy. Understanding possible future evolutions of this fraction under climate policy therefore remains an important challenge for further research.

Given the currently irreducible uncertainties in observationally based estimates of the TCRE, prudent climate policy to hedge against the possibility of a high TCRE is required. However, climate policies should ideally be designed to incorporate new information about the climate response into the ratcheting up of mitigation ambition over the century, as we learn more about the climate response over time. Earth system modelling approaches that more directly parallel the Paris Agreement's goal-driven pledge and review mechanism to end up with stabilized warming at specific thresholds [57] may be more effective ways of using Earth system modelling frameworks to assess what future mitigation pathways might be consistent with ambitious mitigation goals.

## Supplementary Material

Supplementary Information and Figures

## Data accessibility

CMIP5 ESM data can be downloaded from: https://esgf-node.llnl.gov/search/cmip5/. HadCRUT4-CW data are made available at http://www-users.york.ac.uk/~kdc3/papers/coverage2013/series.html. GCP emissions are made available at http://cdiac.ess-dive.lbl.gov/GCP/carbonbudget/2016/. Blended model output for the historical and RCP8.5 simulation is available at http://www-users.york.ac.uk//~kdc3/papers/robust2015/methods.html. Analysis code can be made available from the authors on request.
