# Supplementary material for: The utility of the historical record for assessing the transient climate response to cumulative emissions
Source: Philos Trans A Math Phys Eng Sci. 2018 Apr 2;376(2119):20160449. doi: 10.1098/rsta.2016.0449 (PMC5897822; doi:10.1098/rsta.2016.0449)
Supplement: Supplementary Information and Figures [file rsta20160449supp1.pdf]

Supplementary Information and Figures for "The utility of the historical record for assessing the transient climate response to cumulative emissions"

Authors: Richard J. Millar, Pierre Friedlingstein

To test the similarity of carbon-cycle feedbacks between the 1%/yr and historical simulations, we conduct emulations of the range of ESM response with a simple carbon cycle-climate model (FAIR) [1]. Using ESM-output as targets, we emulate individual ESM simulation with FAIR using the methodology described in the supplementary material of ref [2]. We first fit the thermal climate response parameters to the warming response of a given ESM under the 1%/yr experiment, and then fit the carbon-cycle feedback parameters using the ESM-diagnosed cumulative emissions as a target. This fitting exercise successfully produces a set of simple climate model parameter settings that reproduce the range of response exhibited by the ESM ensemble under the 1%/yr experiment. We then use these 1%/yr fitted parameter settings to diagnose cumulative emissions under the RCP8.5 (Figure S2) and RCP2.6 (Figure S3) experiments (dashed lines) when forced with ESM-specific effective radiative forcing as estimated by ref [3]. Only models for which forcing estimates have been calculated by ref [3] are shown. In most of the ESMs examined, biases in diagnosed emissions using the 1%/yr emulated FAIR parameter settings are typically small over the historical period, but grow substantially under high future emissions scenarios (such as RCP8.5) for which the airborne fraction does not increase as fast as in the ESM simulations, leading to much higher diagnosed cumulative CO_2 emissions than seen in the ESM output or in FAIR emulations of the RCP8.5 scenario.

This difference in emulated carbon-cycle feedbacks between the 1%/yr and RCP experiments is an important caveat to using the method presented in Section 3.2.1 to estimate how the non-CO_2 warming fraction is projected to change in the future by ESMs. Applying this method forward into the future as shown in Figure S4, may potentially lead to an over-estimate of the fraction of warming associated with non-CO_2 forcing in an RCP8.5 like future by underestimating CO_2 induced warming for a given amount of diagnosed cumulative emissions. Given these caveats, the results regarding how the fraction of non-CO_2 warming evolves in RCP2.6 and RCP8.5 scenarios discussed below should be interpreted with caution.

Contrasting the very high emissions future of RCP8.5 [4], which is similar to a business-as-usual pathway, and the deep decarbonisation future under RCP2.6 [5] shows substantial differences between the relative importance of non-CO_2 induced warming to total warming (Figure S4). The ESM ensemble mean non-CO_2 warming fraction is ~23% in 2016, slightly lower than the best-estimate from observations (Figure 4c). Under RCP2.6 scenario the ESM mean decreases to only 15% by the end of the century. Model uncertainty under this scenario is sufficiently large to include zero net contribution of non-CO_2 warming to total warming at the end of the century. In contrast, under RCP8.5 the ensemble mean fraction increases slightly to 31% by the end of the century. These two scenarios represent just two potentially plausible future emissions scenarios that were selected as they approximately spanned the range of scenarios available in the literature [6].

Whilst the absolute fractional contribution of non-CO_2 induced warming is uncertain, the relative changes between 2020 and the end of the century are more constrained in both RCP8.5 and RCP2.6 ESM simulations. The fraction increases by 8% in the mean (-3% to +36%; min-max range) across the ensemble in RCP8.5 and decreases by 7% (-26% to +14%) in RCP2.6, suggesting that contributions from non-CO_2 could be expected to decrease under very ambitious climate policy focused on both CO_2 and non-CO_2 pollutants. Due to the limited sample size of available ESMs, the RCP2.6 and RCP8.5 distributions of non-CO2 warming fraction at the time of crossing the 1.5°C are not statistically distinguishable (at a 5% level under a two-sided Student-t test), but relative changes by 2100 are statistically distinguishable between the two scenarios.

References

1. Millar RJ, Nicholls ZR, Friedlingstein P, Allen MR. 2017 A modified impulse-response representation of the global near-surface air temperature and atmospheric concentration response to carbon dioxide emissions. *Atmos. Chem. Phys.* **17**, 7213–7228. (doi:10.5194/acp-17-7213-2017)
2. Millar RJ *et al.* 2017 Emission budgets and pathways consistent with limiting warming to 1.5 °C. *Nat. Geosci.*, 1–8. (doi:10.1038/ngeo3031)
3. Forster PM, Andrews T, Good P, Gregory JM, Jackson LS, Zelinka M. 2013 Evaluating adjusted forcing and model spread for historical and future scenarios in the CMIP5 generation of climate models. *J. Geophys. Res. Atmos.* **118**, 1139–1150. (doi:10.1002/jgrd.50174)
4. Riahi K, Rao S, Krey V, Cho C, Chirkov V, Fischer G, Kindermann G, Nakicenovic N, Rafaj P. 2011 RCP 8.5-A scenario of comparatively high greenhouse gas emissions. *Clim. Change* **109**, 33–57. (doi:10.1007/s10584-011-0149-y)
5. van Vuuren D *et al.* 2011 RCP 2.6: exploring the possibility to keep global mean temperature increase below 2°C. *Clim. Change* **109**, 95–116. (doi:10.1007/s10584-011-0152-3)
6. Moss RH *et al.* 2010 The next generation of scenarios for climate change research and assessment. *Nature* **463**, 747–56. (doi:10.1038/nature08823)
7. Rohde R, Muller R, Jacobsen R, Muller E, Groom D, Wickham C. 2012 A New Estimate of the Average Earth Surface Land Temperature Spanning 1753 to 2011. *Geoinformatic Geostatistics An Overv.* **1**, 1–7. (doi:http://dx.doi.org/10.4172/gigs.1000101)
8. Le Quéré C *et al.* 2017 Global Carbon Budget 2017. *Earth Syst. Sci. Data Discuss.* **2017**, 1–79. (doi:10.5194/essd-2017-123)

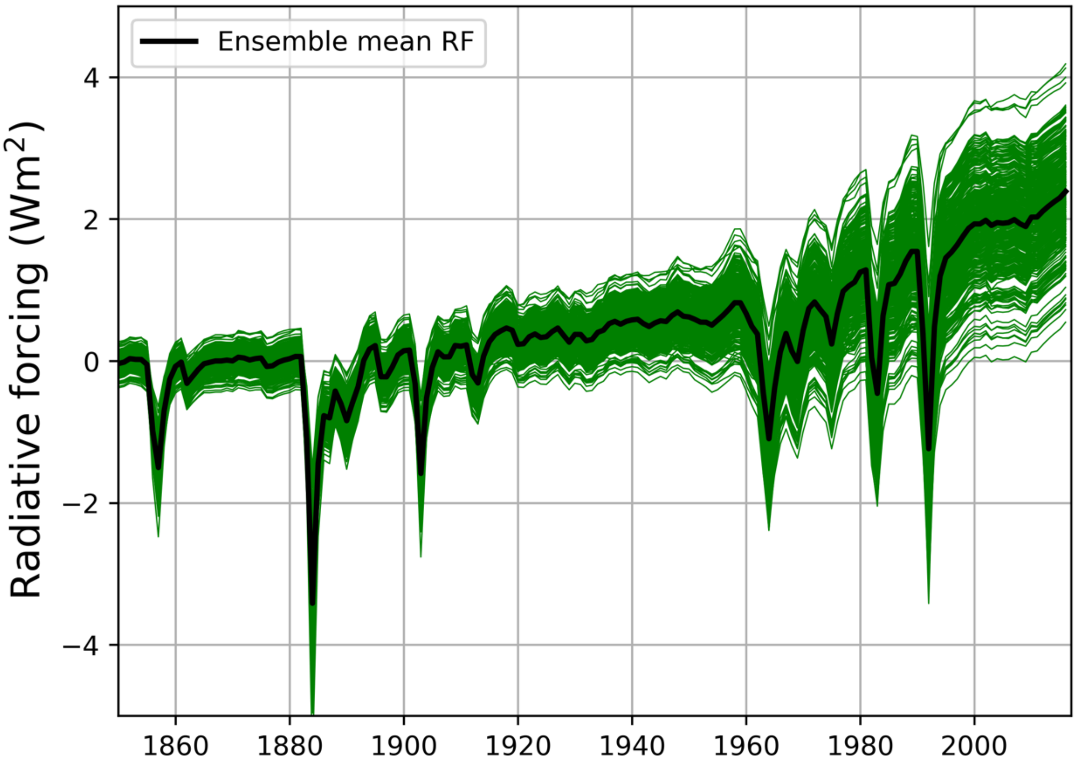

Figure S1: Ensemble of total (anthropogenic + natural) effective radiative forcing over the historical period. Green lines represent samples from assessed uncertainties in components of radiative forcing. Thick black line shows the ensemble mean forcing.

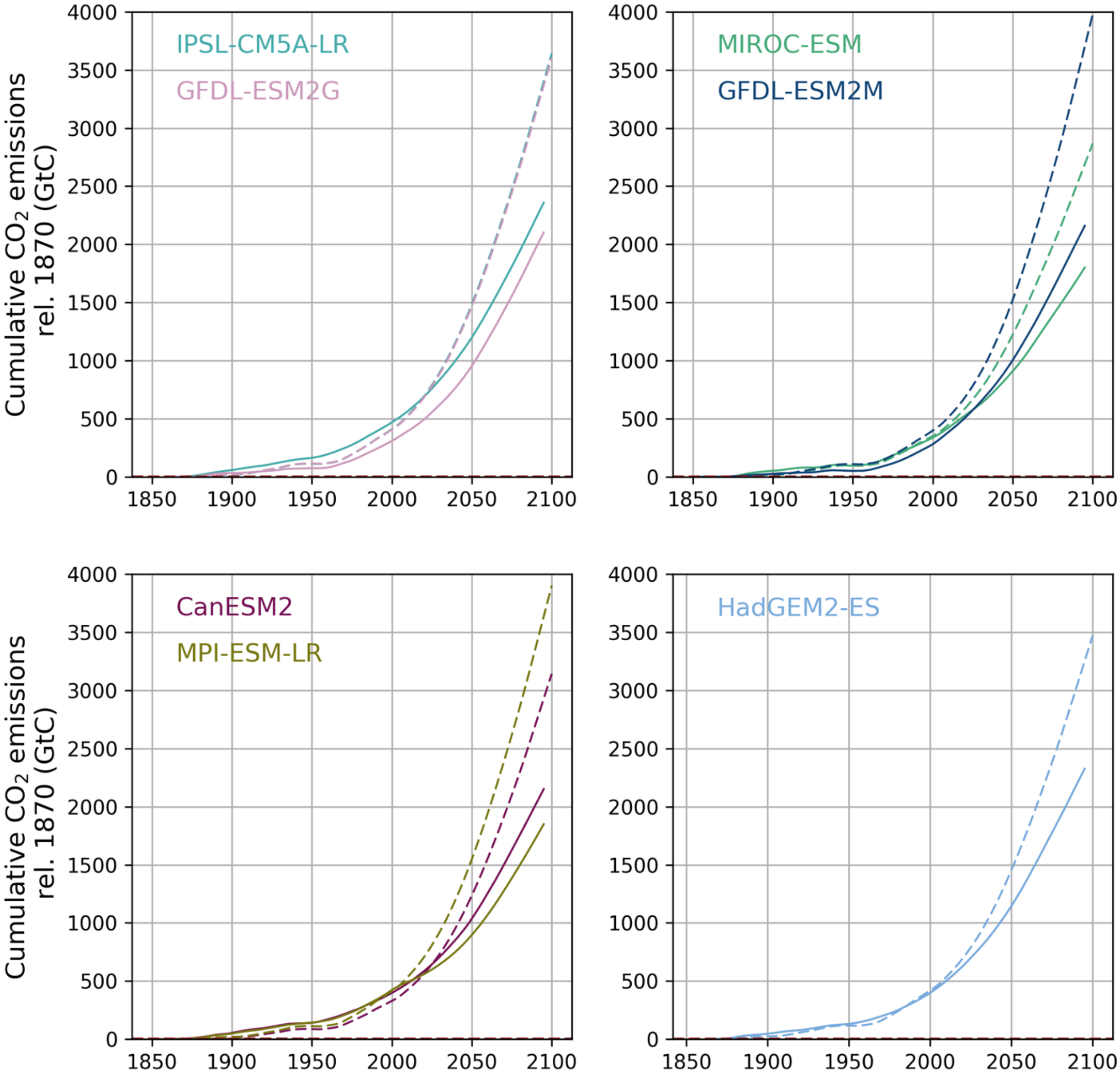

Figure S2: Diagnosed cumulative emissions in the RCP8.5 scenario from ESMs (solid lines) and from versions of the FAIR simple climate model with parameters fit to emulate the relationship between forcing, diagnosed emissions and warming in the ESM's 1%/yr CO_2 increase experiment (dashed lines).

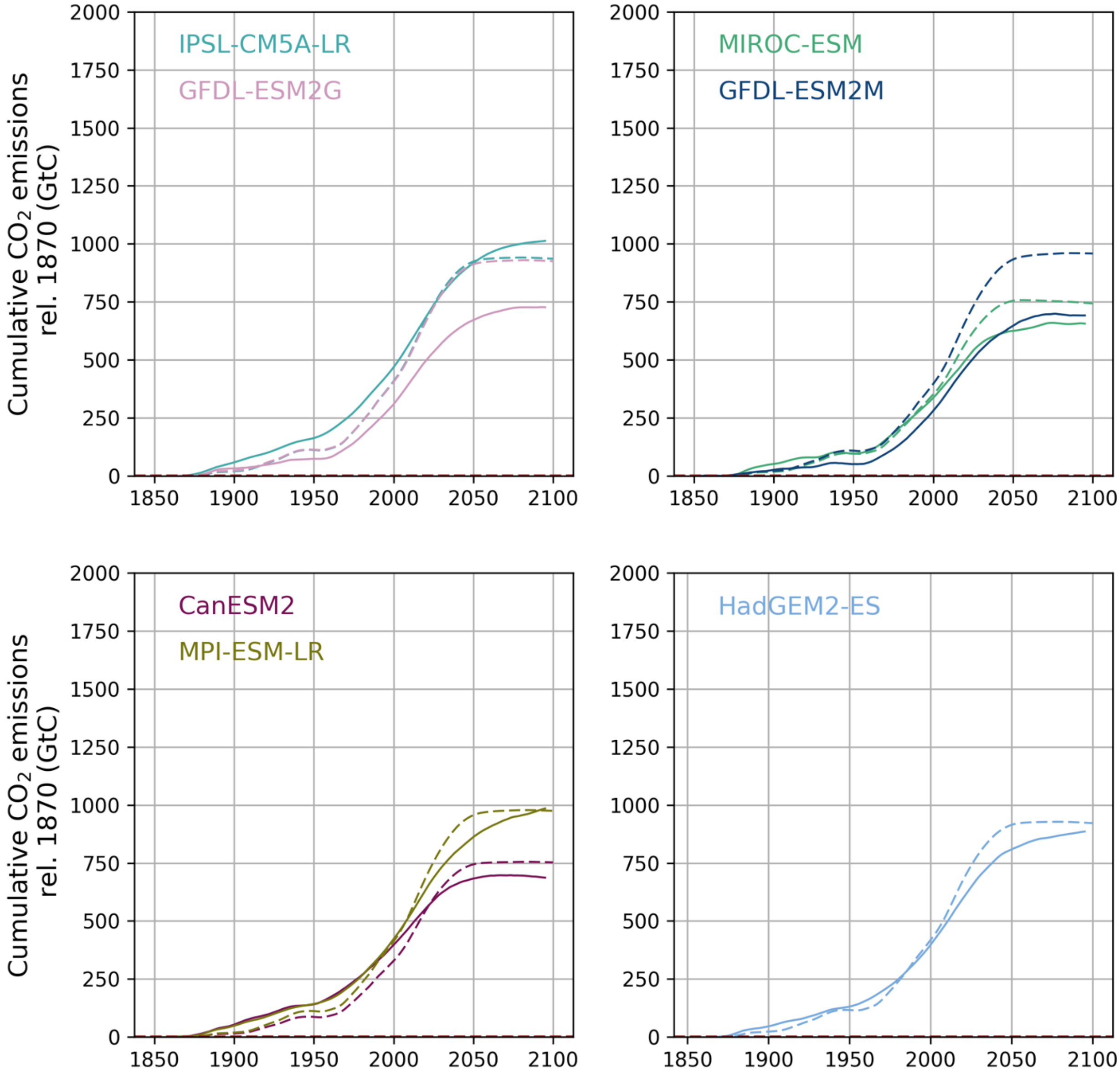

Figure S3: As for Figure S2 but for the RCP2.6 scenario.

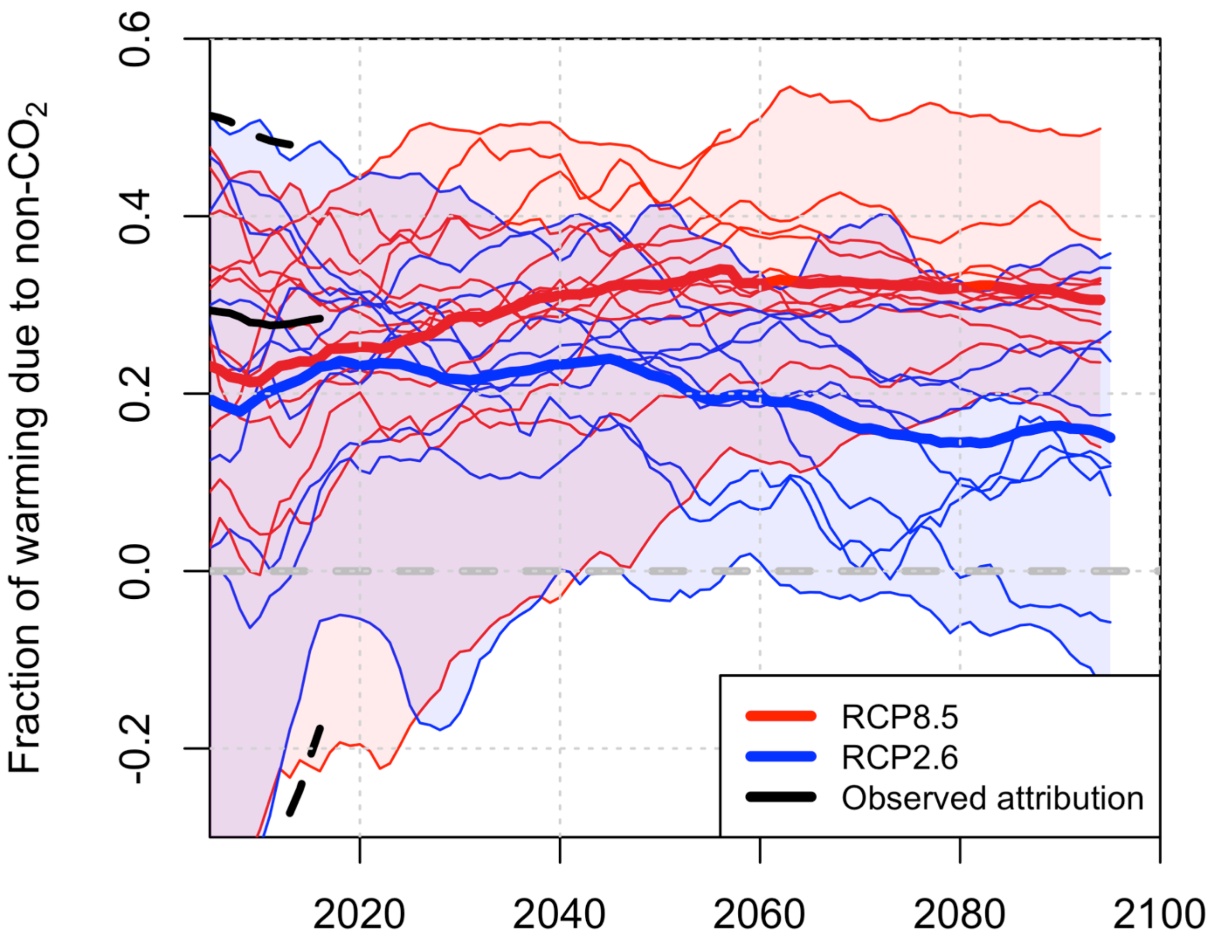

Figure S4: Future evolution of the fractional contribution of non-CO_2 warming to total warming under the RCP2.6 (blue) and RCP8.5 (red) scenarios. Multi-model means are shown by thick solid lines (thin solid lines are individual ensemble members) with ensemble spread indicated by shading. Black lines indicate the best-estimate (solid) and 5/95 percentile (broken) attributed non-CO_2 warming fractions up to the end of 2016.

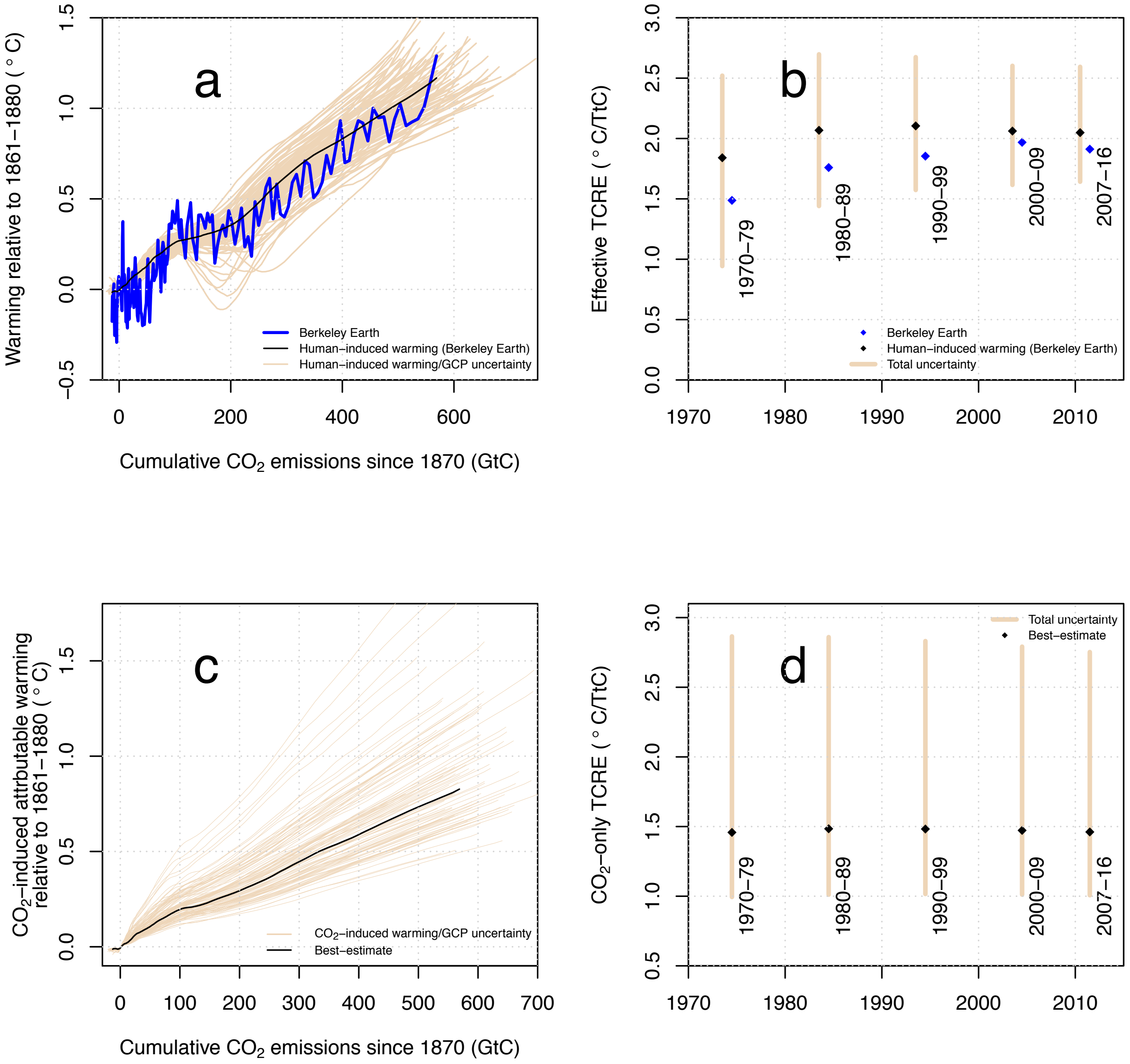

Figure S5: As for Figure 1 of the main text, but for the Berkeley Earth temperature dataset [7]. Unlike for HadCRUT4-CW, no ensemble of observational uncertainty is provided so observational uncertainty is left out of the uncertainty assessment.

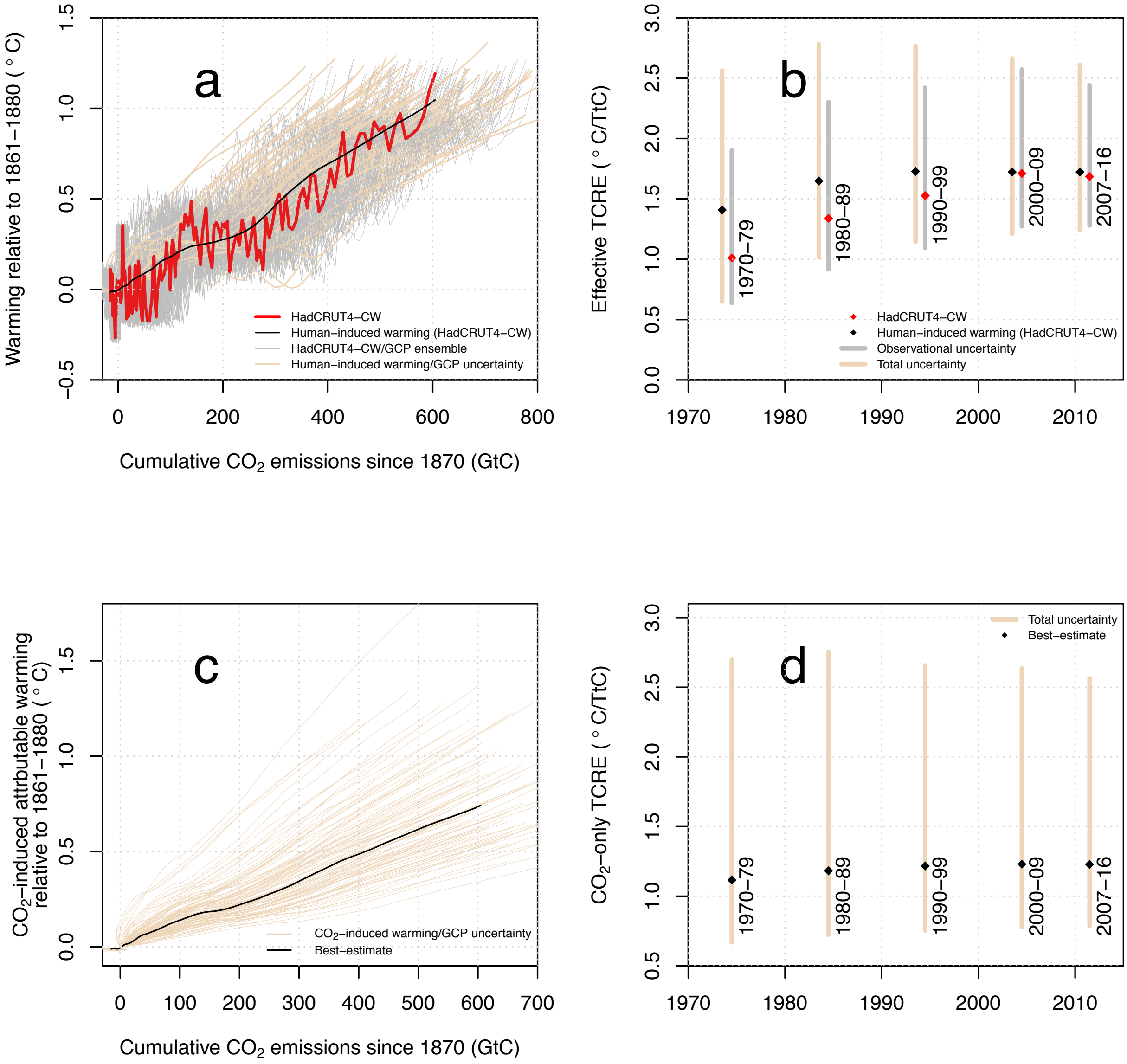

Figure S6: As for Figure 1 of the main text, but for the 2017 GCP emissions dataset [8].
